# Supplementary material for: Repurposing 9-Aminoacridine as an Adjuvant Enhances the Antimicrobial Effects of Rifampin against Multidrug-Resistant Klebsiella pneumoniae
Source: Microbiol Spectr. 2023 Apr 10;11(3):e04474-22. doi: 10.1128/spectrum.04474-22 (PMC10269603; doi:10.1128/spectrum.04474-22)
Supplement: Supplemental file 1 — Fig. S1 to S7 and Tables S1 to S3. Download spectrum.04474-22-s0001.pdf, PDF file, 2.3 MB [file spectrum.04474-22-s0001.pdf]

Supplementary Material

Table S1. Bacterial strains used in this study

Strains	Resistant pattern	Source
**Gram-negative**		
K. pneumoniae		
ATCC 700603	Type strain, Non-MDR	ATCC
ATCC 4352	Type strain, Non-MDR	ATCC
ATCC 10031	Type strain, Non-MDR	ATCC
KPWANG	XDR	Clinical isolated(1)
KPLUO	XDR	Clinical isolated(1)
LH2020	PDR	Clinical isolated(1)
P. aeruginosa		
PAO1(ATCC 15692)	Type strain, Non-MDR	ATCC
PA2530	XDR	Clinical isolated
PA6930	XDR	Clinical isolated
A. baumannii		
ATCC 19606	Type strain, Non-MDR	ATCC
AB1095	XDR	Clinical isolated
E. coli		
ATCC 25922	Type strain, Non-MDR	ATCC
Y0064	XDR	Clinical isolated
Y9592	XDR	Clinical isolated
**Gram-positive**		
S. aureus		
ATCC 43300	Type strain, MRSA	ATCC
USA300	Type strain, MRSA	ATCC
ATCC 29213	Type strain, MSSA	ATCC
S. epidermidis		
RP62A(ATCC 35984)	Type strain, Non-MDR	ATCC
ATCC 12228	Type strain, Non-MDR	ATCC
E. faecalis		
ATCC 29212	Type strain, Non-MDR	ATCC
E. faecium		
U101	VRE	Clinical isolated(2)

ATCC, American Type Culture Collection.MRSA, Methicillin-resistant *S.aureus*. MSSA, Methicillin-Sensitive *S.aureus*.VRE, vancomycin-resistant *E. faecium.*

References

1. She P, Liu Y, Xu L, Li Y, Li Z, Liu S, Hussain Z, Wu Y. 2022. SPR741, Double- or Triple-Combined With Erythromycin and Clarithromycin, Combats Drug-Resistant Klebsiella pneumoniae, Its Biofilms, and Persister Cells. Frontiers In Cellular and Infection Microbiology 12:858606.
2. She P, Wang Y, Li Y, Zhou L, Li S, Zeng X, Liu Y, Xu L, Wu Y. 2021. Drug Repurposing: In vitro and in vivo Antimicrobial and Antibiofilm Effects of Bithionol Against Enterococcus faecalis and Enterococcus faecium. Frontiers In Microbiology 12:579806.

Table S2. Antibacterial susceptibility of 9-AA in the presence of PGN against ATCC 700603

	9-AA	+ 10 μg/mL PGN	+ 20 μg/mL PGN
MIC (μg/mL)	16	16	16
MBC (μg/mL)	64	64	64

Table S3. IC50 (μg/mL) of 9-AA ,9-AA(L) and 9-AA analogs to human cell lines

Cell lines	Liver		Kidney		Nerve	
	LO2	HepG2	HK2	786-O	HMC3	U251
9-AA	7.22	5.95	11.88	5.81	7.5	8.34
9-AA(L)	8.01	11.54	18.64	12.86	8.55	10.15
ethacridine	4.14	19.71	36.93	2.06	2.04	3.21
euflavine	<2	6.08	3.76	3.11	3.76	5.07

Fig S1

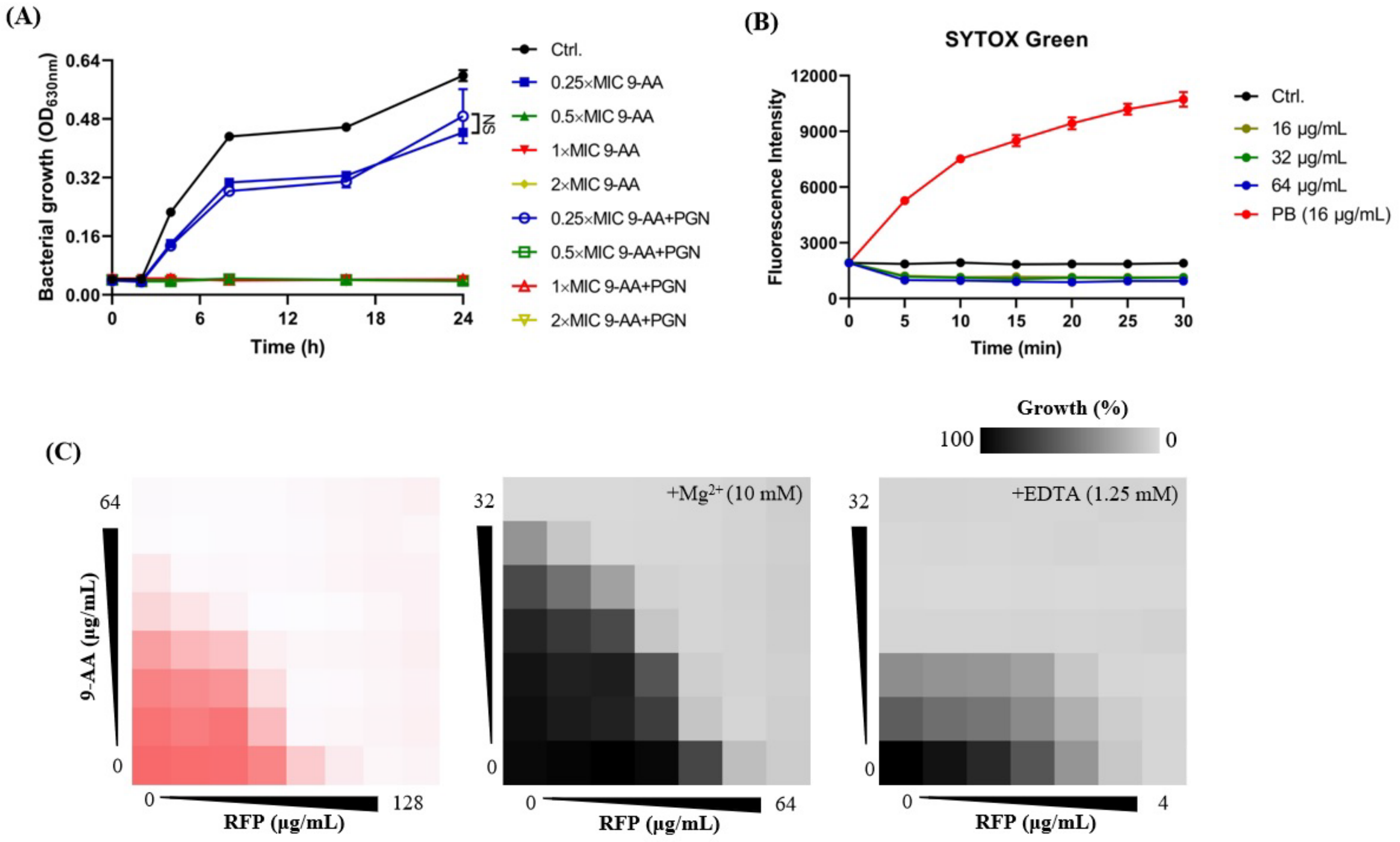

Figure S1 Bacterial cell wall component-disrupting activity of 9-AA against *K. pneumoniae* ATCC 700603. (A) Time growth curves of 9-AA in the presence of purified PGN. (B) The effect of 9-AA on the bacterial cytoplasmic membrane was assessed by SYTOX Green with increasing concentrations of 9-AA and 16 μg/mL polymyxin B (PB) as positive control. (C) Representative checkerboard images of 9-AA and RFP in the presence of Mg^{2+} or EDTA. The leftmost checkerboard shared the same image as Figure 1B.

Fig S2

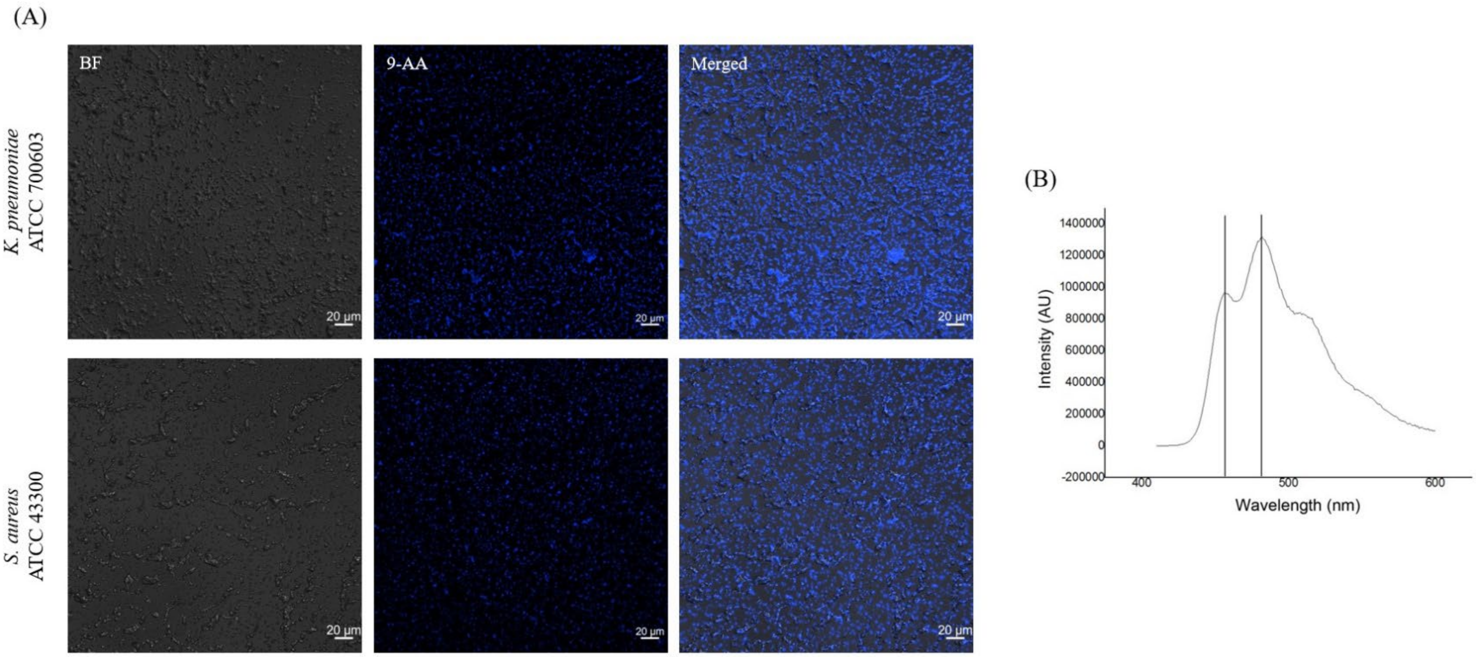

Figure S2 Fluorescence characteristics of 9-AA. (A) Confocal images of *K. pneumoniae* ATCC 700603 and *S. aureus* ATCC 43300 exposed to 32 µg/mL 9-AA for 30 min. (B) Fluorescence emission spectra of 9-AA with excitation wavelengths at 390 nm and emission wavelengths ranging from 410–600 nm.

Fig S3

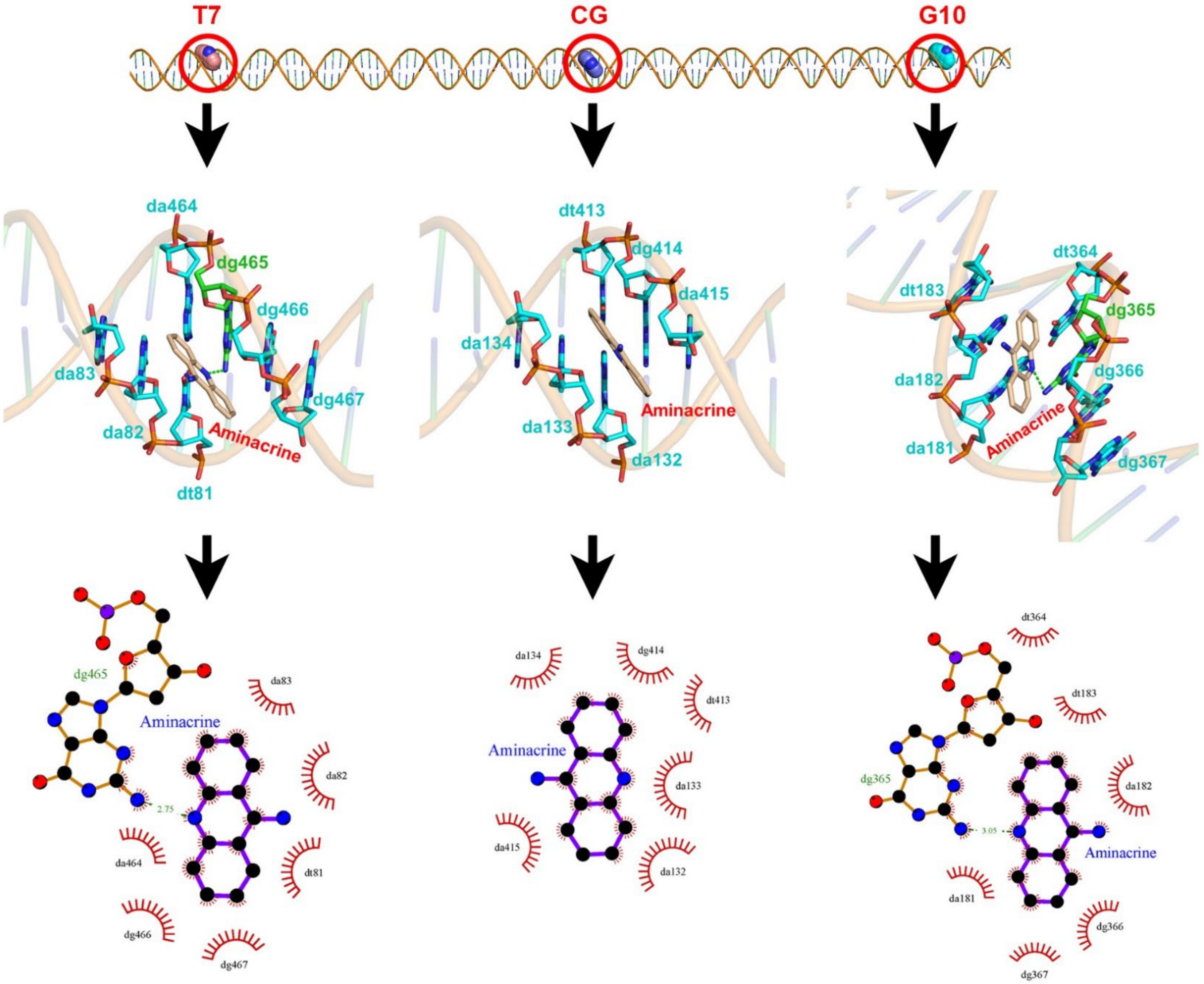

Figure S3 Schematics of 9-AA binding to DNA fragments (T7, CG, G10). The first row shows schematics of the DNA-binding site location. The second row shows three-dimensional schematics of 9-AA binding to the DNA fragments of T7, CG, and G10. Green lines indicate hydrogen bonds, and red gears indicate hydrophobic interactions. The third row shows two-dimensional schematics of 9-AA binding to DNA (T7, CG, G10), and the green dotted lines represent the hydrogen bond.

Fig S4

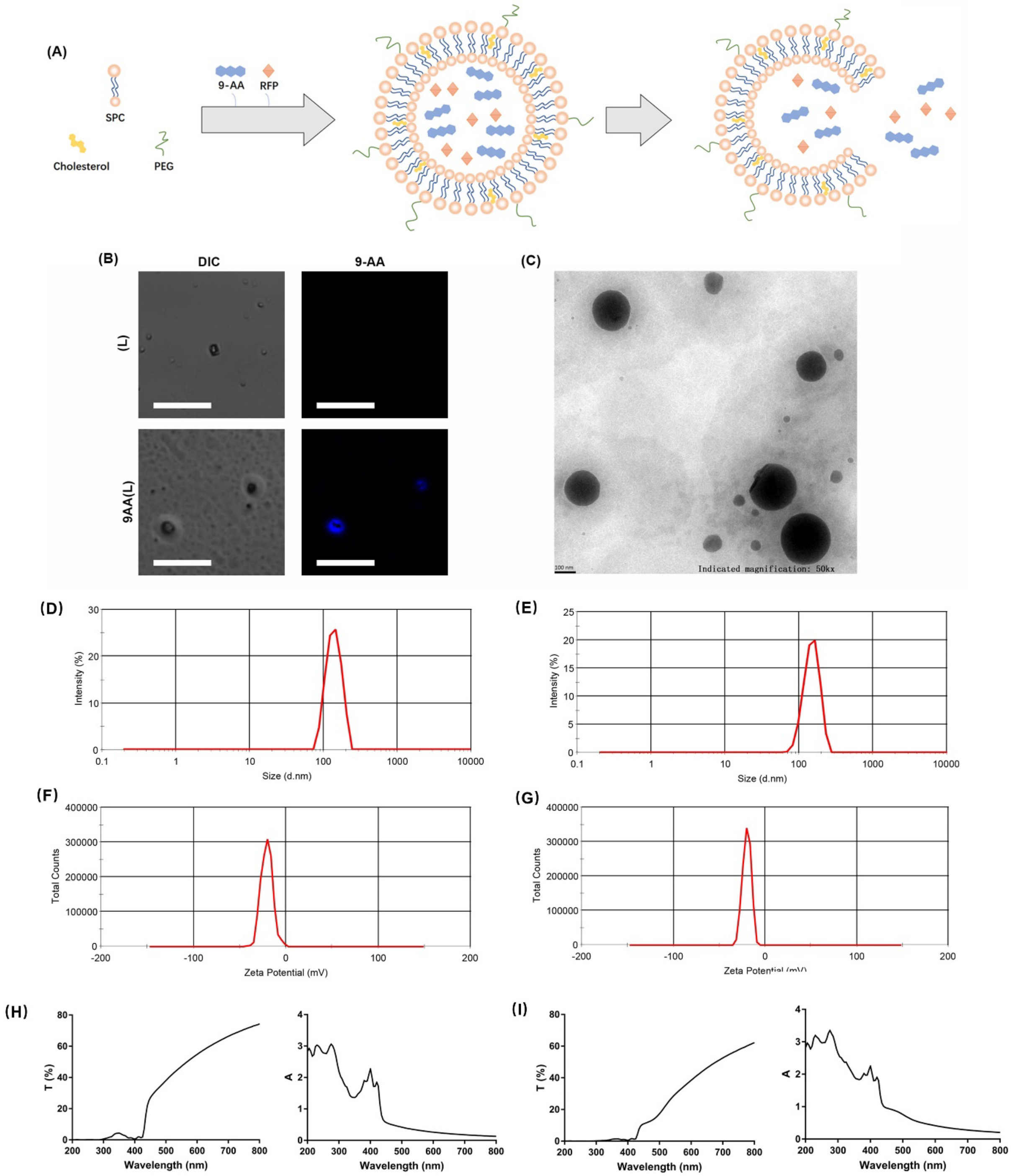

Figure S4 Characterization of 9-AA(L) and (9-AA+RFP)(L). (A) Schematic of the preparation and drug release of liposomes. (B) CLSM images of empty vector (L) and 9-AA(L). Scale bar: 20 µm. (C) TEM images of 9-AA(L). Scale bar: 100 nm. Intensity distribution data of (D) 9-AA(L) and (E) (9-AA+RFP)(L), respectively. Zeta potentials of (F) 9-AA(L) and (G) (9-AA+RFP)(L). UV–vis absorption and transmission spectra of (H) 9-AA(L) and (I) (9-AA+RFP)(L).

Fig S5

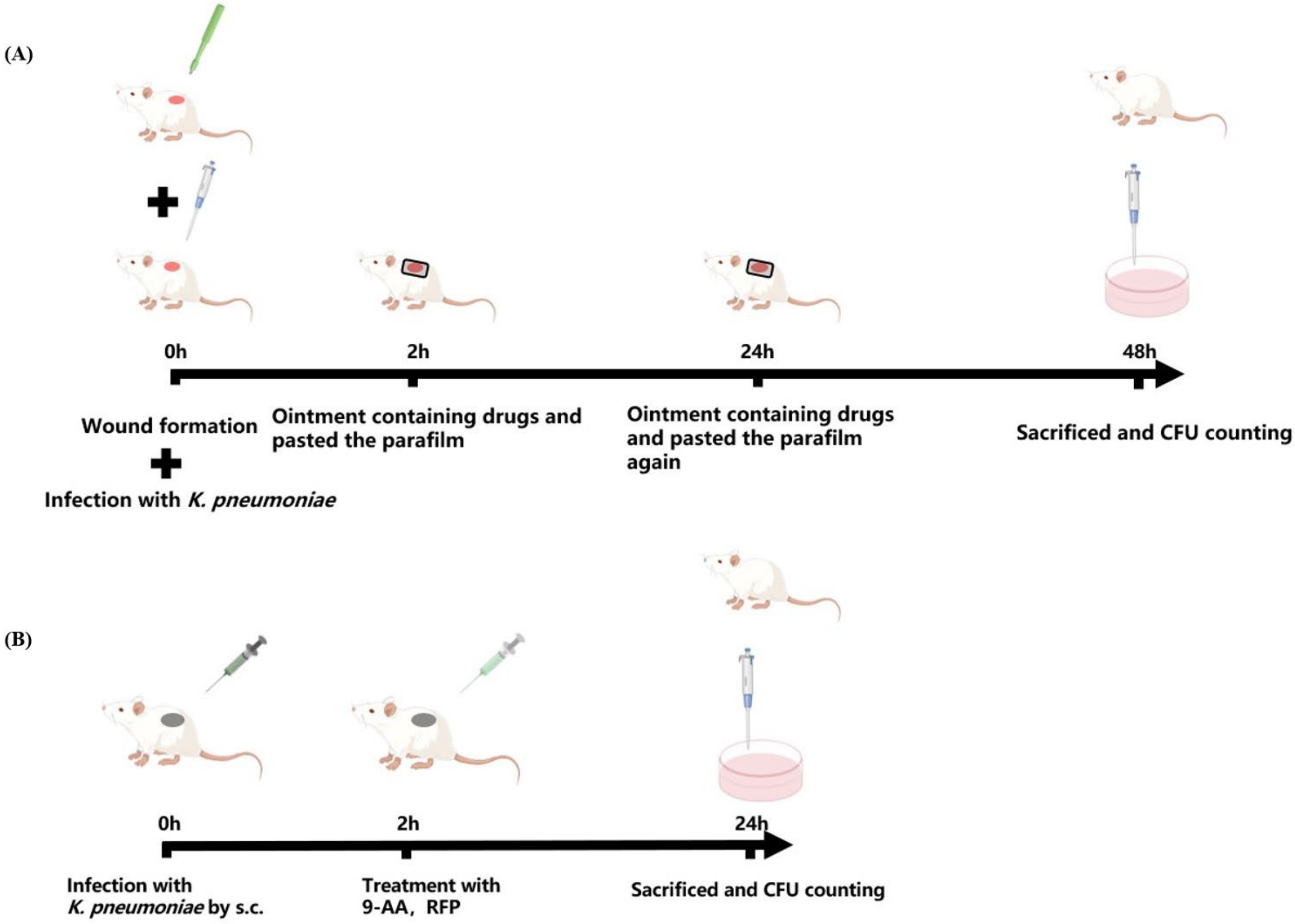

Figure S5 Flowchart of *K. pneumoniae*-related skin and soft tissue infection experiments. (A) The wound infection model. (B) The subcutaneous skin abscess model.

Fig S6

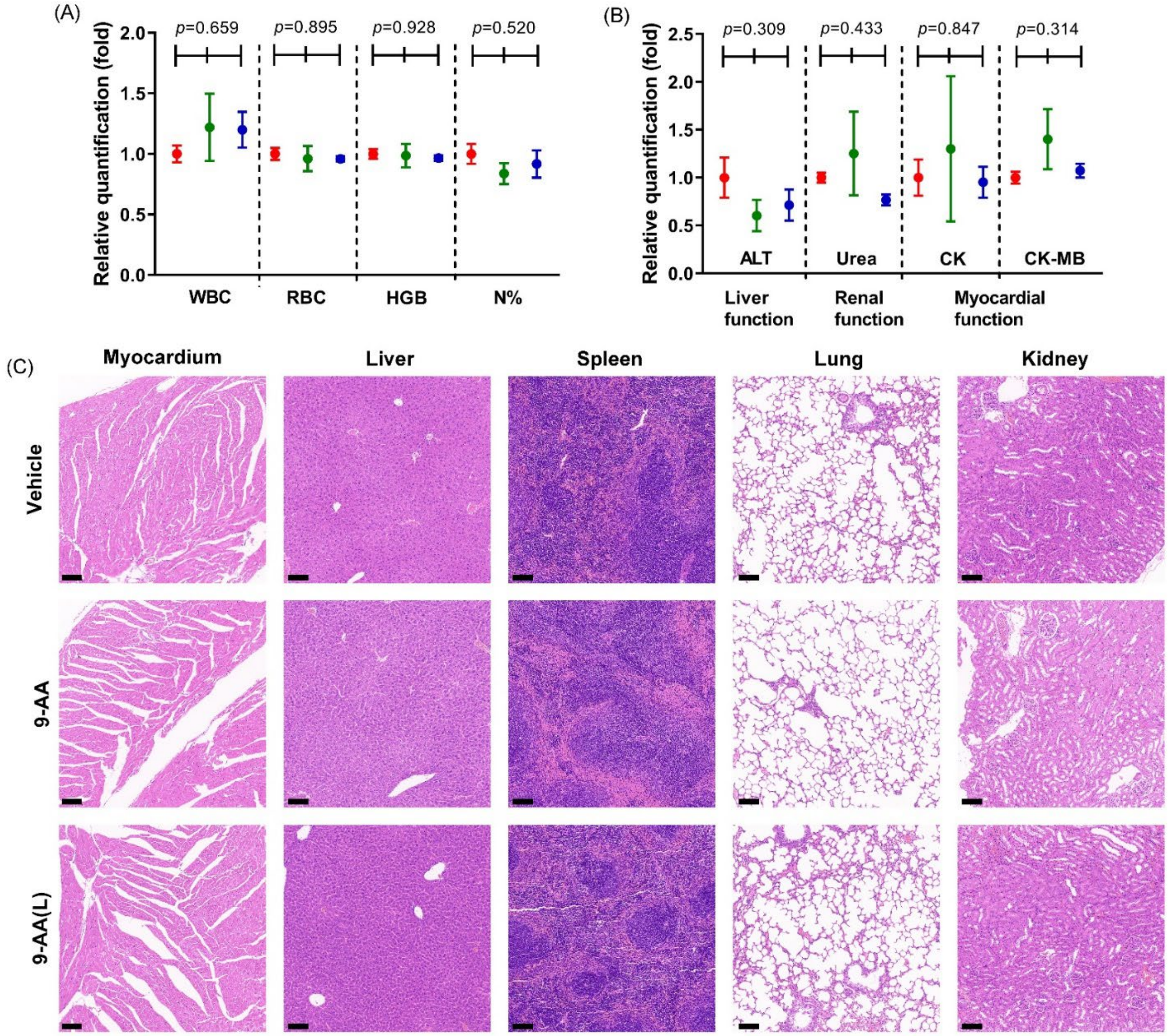

Figure S6 *In vivo* safety assessments of 15 mg/kg 9-AA and 15 mg/kg 9-AA(L). (A) Analysis of routine blood parameters: WBC, white blood cell. RBC, red blood cell. HGB, hemoglobin quantification. PLT, platelets. N%, percentage of neutrophils in whole blood. The vehicle, 9-AA and 9-AA(L) groups are represented in red, green and blue respectively. (B) Biochemical index analysis. ALT, alanine aminotransferase. Urea, urea nitrogen. CK, creatine kinase, CK-MB isoenzyme. (C) H&E staining of myocardium, liver, spleen, lung, and kidney. n=5/group. Scale bars: 200 μm. All data are expressed as the means ± SDs.

Fig S7

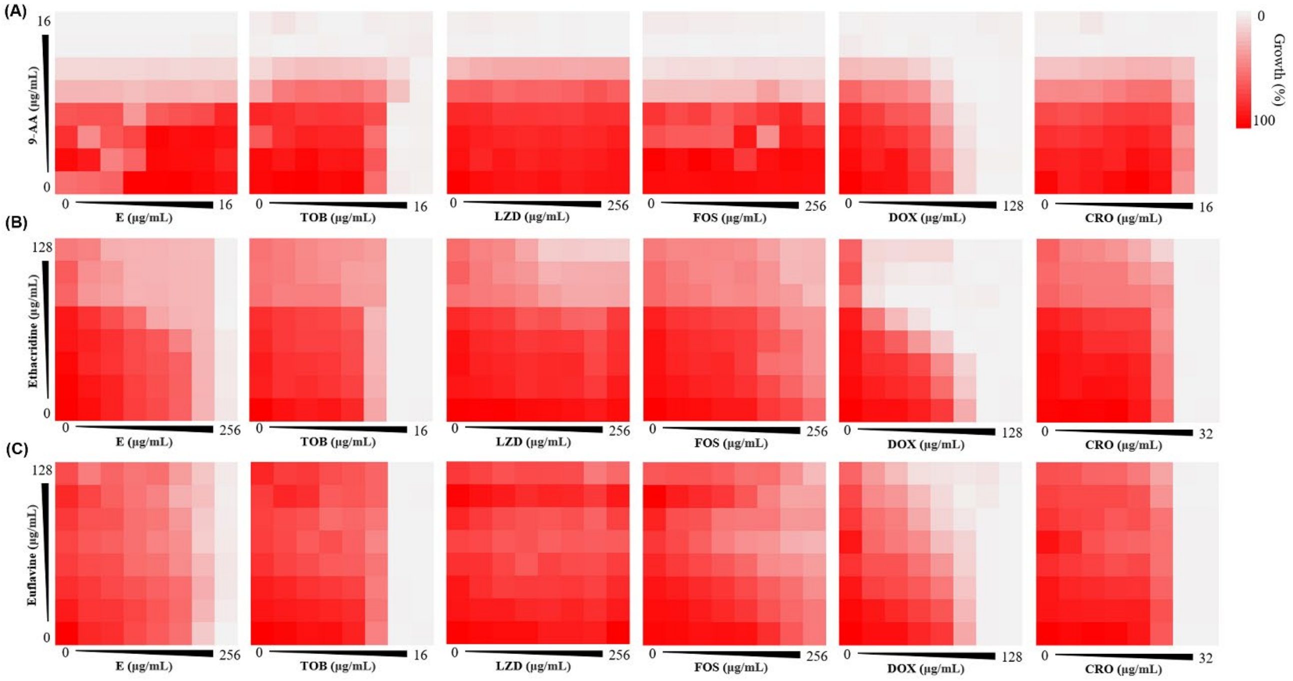

Figure S7 The representative checkerboard images of drug combination between 9-AA and 9-AA analogs with conventional antibiotics against *K. pneumoniae*. E = erythromycin, TOB = tobramycin, LZD = linezolid, FOS = fosfomycin, DOX = doxycycline, CRO = Ceftriaxone.
